# First case of human peritoneal cysticercosis mimicking peritoneal carcinosis: necessity of laparoscopy and histologic assessment for the correct diagnosis

**DOI:** 10.1099/jmmcr.0.005097

**Published:** 2017-06-08

**Authors:** Martina Rudelius, Klaus Brehm, Martin Poelcher, Christoph Spinner, Andreas Rosenwald, Clarissa Prazeres da Costa

**Affiliations:** ^1^​ Institute of Pathology, University of Wuerzburg and CCC Mainfranken, Josef-Schneider-Straße 2, 97080 Würzburg, Germany; ^2^​ Institute of Pathology, University of Duesseldorf, Moorenstrasse 5, 40225 Düsseldorf, Germany; ^3^​ Institute of Hygiene and Microbiology, University of Wuerzburg, Josef-Schneiderstrasse 2, 97070 Würzburg, Germany; ^4^​ Frauenklinik am Rotkreuzklinikum München, 80634 München, Germany; ^5^​ Department of Medicine II, University Hospital Klinikum rechts der Isar, 81675 München, Germany; ^6^​ Institute of Medical Microbiology, Immunology and Hygiene, Technische Universität München (TUM), Ismaningerstrasse 25, 80333 München, Germany

**Keywords:** human cysticercosis, peritoneal

## Abstract

**Introduction.** Correct diagnosis of peritoneal infectious disease can be extremely difficult due to non-specific clinical features. Thus, careful assessment with thorough histopathological work-up is essential. Here, we report the first case of human peritoneal cysticercosis mimicking peritoneal carcinosis.

**Case presentation.** The patient presented with recurring ascites and a tumour in the Douglas cavity accompanied by elevated tumour markers. There were no signs of systemic infection. On laparoscopy, the tumour was resected completely. Histology revealed a granulomatous reaction and a diagnosis suspicious of tuberculosis was made. Only after additional sections, avital cestode-fragments were visible and *Taenia martis* DNA was detected. Further staging by computerized tomography scan of the lung and brain turned out negative and the patient recovered quickly.

**Conclusion.** Laparoscopy and histopathological examination can be extremely helpful for correct diagnosis and management in uncertain recurrent ascites. This case clearly demonstrates that orphan infectious diseases should also be considered. Only complete histopathological examination with serial sections and additional molecular testing can lead to the appropriate diagnosis.

## Abbreviations

CRP, C-reactive protein; MRI, magnetic resonance imaging.

## Introduction

Peritoneal infectious disease is difficult to diagnose. Elevated C-reactive protein (CRP), eosinophilia or signs of systemic infectious disease can lead to the correct diagnosis. However, often those signs are missing, thus delaying appropriate treatment.

In our case, not only were specific symptoms of an infectious disease, such as fever, night sweats or weight loss, missing, but also some observations such as recurrent ascites, a tumour in the Douglas cavity and elevated laboratory tumour markers prompted the diagnosis of peritoneal carcinosis. Only laparoscopy and complete histological work-up led to the diagnosis of peritoneal cysticercosis. Therefore, we want to draw attention to the importance of complete histopathological work-up with specific stains for a correct diagnosis. It is also important to broaden the spectrum of differential diagnosis of peritoneal (infectious) disease, as this is the first case of human peritoneal cysticercosis among the three reported cases of human *Taenia martis* cystercercosis worldwide.

## Case eport

A 36-year-old woman presented to her gynecologist with recurring, asymptomatic ascites, which progressed slightly over a period of 8 weeks. There were no signs of systemic infectious disease with normal CRP, normal white blood cell count and no evidence of eosinophilia. However, laboratory tumour marker Cancer Antigen 125 (CA125) was elevated at initial presentation with normal carcinoembryonic antigen (CEA), Cancer Antigen 19.9 (CA19.9) and Cancer Antigen 72.4 (CA72.4). Medical ultrasound of the abdomen did not reveal any further findings, but with magnetic resonance imaging (MRI) a tumour (1.6×1.6 cm) could be detected in the Douglas cavity.

**Fig. 1.**
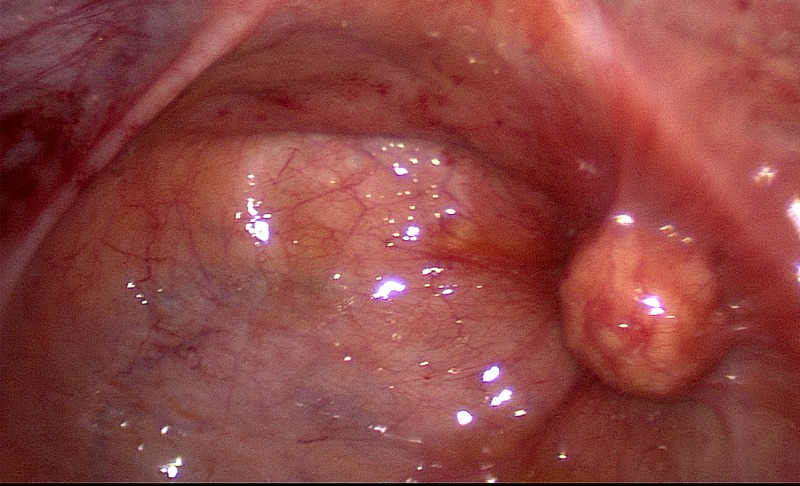
Intraoperative picture showing a well-circumscribed 1.6×1.6×1.6 cm tumour in the Douglas cavity.

The patient wanted to become pregnant and was alarmed by the initially elevated laboratory tumour markers. Therefore, the decision for laparoscopy was made. Abdominal laparoscopy was performed, and a sharply demarcated tumour with a smooth surface was detected within the Douglas cavity at the posterior cervical wall. The tumour could be resected completely (Fig. 1) and was given for pathologic assessment.

## Investigations

On gross examination, a 1.6×1.6×1.6 cm, circumferential tumour with a white to yellow cut surface was visible.

**Fig. 2.**
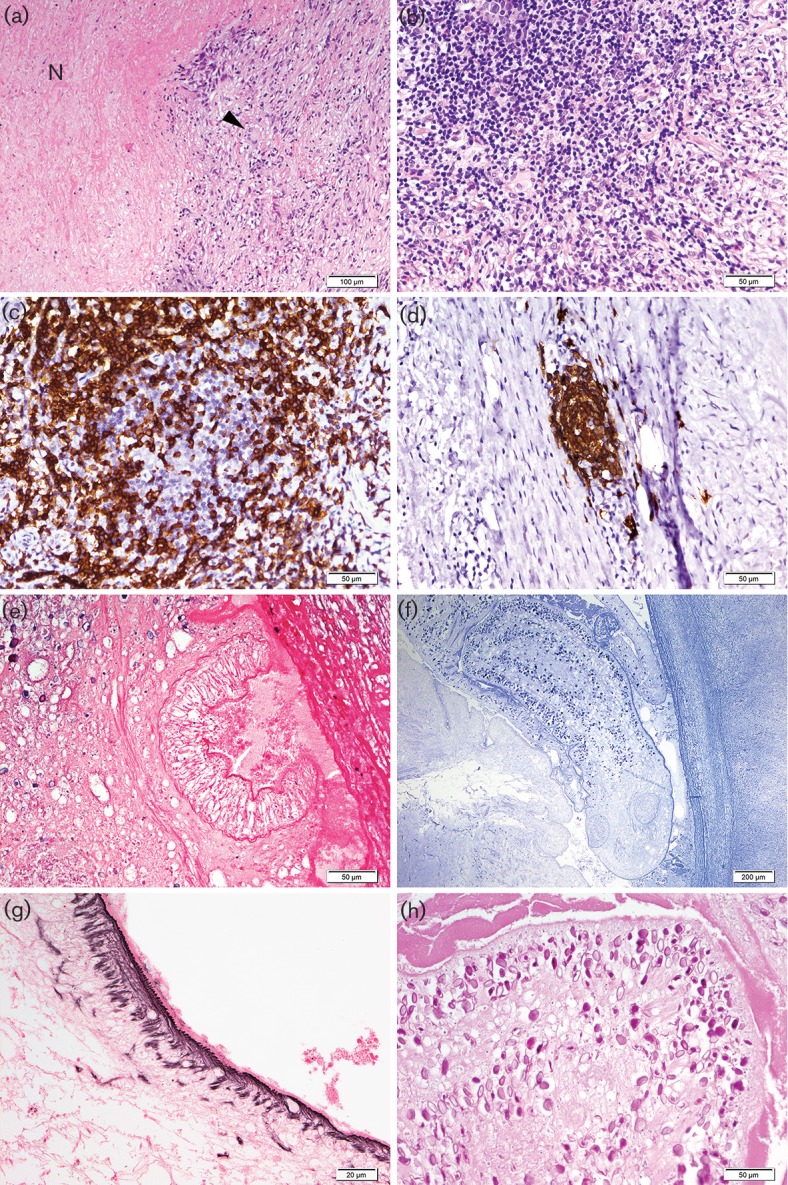
(a) Granulomatous inflammation with central necrosis (N) and epitheloid cells with few Langhans giant cells (h and e). (b) Nodular lymphocytic infiltrate with small mature lymphocytes (h and e), which is composed of a mixture of CD5-positive T-lymphocytes (c) and CD20-positive B-lymphocytes (d). (e and f) In the central necrosis a larva could be detected with suckers, (e) a two-layered tegmentum (g) and calcified corpuscles (h).

Microscopically, initial pathologic analysis revealed sclerosis and a granulomatous reaction with a central necrosis; however, no well-formed granulomas could be identified. Further assessment for infection with *Mycobacterium* turned out negative, and the case was sent to the national reference centre of haematopathology in Wuerzburg (Germany) to rule out a lymphoma with a granulomatous reaction. Serial sections were performed with almost complete sectioning of the tumour. Histopathology assessment revealed a tumour mass with central necrosis and aggregates of epitheloid cells with intermingled multinucleated giant cells of Langhans type in the periphery (Fig. 2a). However, well-formed granulomas characteristic of mycobacterial infection were not visible. There was no striking increase of eosinophilic granulocytes, which could have prompted the diagnosis of a parasitic infection. Sparse cytomorphological mature lymphoid cells turned out normal with a predominance of T-lymphocytes (Fig. 2b–d). Only after complete sectioning of the material, slightly corpuscular avital material in the centre was detected besides the amorphous necrotic eosinophilic material. The material resembled a larva (Fig. 2e, f) with an eosinophilic tegmentum (Fig. 2g) and two layers of smooth muscle bundles. The internal structure was composed of a loose matrix with calcaneus bodies (Fig. 2h).

After detection of the degenerated larva, diagnosis of a helminthic parasite infestation was confirmed by microbiological analysis. Nucleic acid testing with pan-taeniid cestode-specific primers directed against 12S mitochondrial rDNA [1] was performed. The sequence alignment revealed 99 % identity to deposited nucleotide sequences of *T. martis* (ENA accession number LT837855). Interestingly, ELISA for *Echinococcus granulosus* was positive – very likely due to antigen cross-reactivity within the taeniid cestode family.

## Diagnosis

Peritoneal manifestation of *T. martis* cysticercosis.

## Treatment

After diagnosis of *T. martis* cysticercosis, the patient was staged by computed tomography (CT) and MRI and no further cysts were revealed in the lung or brain. As minimal manifestation could have been missed, the patient was treated with 400 mg albendazole twice daily for 4 weeks.

## Outcome and follow-up

The patient recovered quickly from surgery and showed no symptoms of systemic infection.

## Discussion

Our purpose is to stress the benefit of laparoscopy and histopathological assessment for diagnosis of infectious disease of the peritoneum in patients presenting with non-specific symptoms. Especially, a complete histological work-up and specific stains can be essential and provide the clues for a correct diagnosis.

Our patient presented with peritoneal fluid, a tumour in the Douglas cavity and elevated CA125. There were no signs of systemic infection, such as eosinophilia, elevated CRP or fever.

These symptoms can result in a broad spectrum of differential diagnoses like peritoneal carcinosis, lymphangioma, gastrointestinal stroma tumour, inflammatory pseudotumour or *Mycobacterium tuberculosis* infection. In our case, the manifestation of a tumour was favoured over the diagnosis of an infectious disease. It is known that abdominal tuberculosis can be difficult to diagnose with possible negative bacteriological results and non-specific symptoms including elevated CA125 [2]. After discussion with the patient, the decision for laparoscopy was made.

A sharply demarcated tumour was resected and given for histopathological analysis. Granulomatous reaction with loose aggregates of epitheloid histiocytes with intermingled mature lymphocytes was found; however, no well-formed granulomas were detected. The differential diagnosis of granulomatous reaction is long, including tuberculosis, foreign body reaction, sarcoidosis, helmithic infection or lymphoma (especially non-Hodgkin lymphoma of the T-cell lineage or Hodgkin lymphoma). No mycobacterial DNA could be identified, and the material was sent to the reference centre of haematopathology to rule out lymphoma. Lymphocytes were mature and composed of a mixed population of B- and T-lymphocytes with a predominance of T-lymphocytes, and no Hodgkin or Reed Sternberg cells were visible, so there was no indication of lymphoma. Foreign body material could not be detected, and no fungi-like *Histoplasma* or *Pneumocystis* could be identified by specific stains [periodic acid Schiff (PAS) and Grocott stain]. There was no increase in eosinophilic granulocytes; however, to rule out a helminth infection, serial sections of the tumour were performed. Only after almost complete sectioning of the tumour an avital degenerated larva indicative of a helminthic parasite infestation could be detected in the central necrosis. Therefore, this case clearly demonstrates that only after step by step pathologic work-up the correct diagnosis could be rendered.

Despite being instructive for proceeding with clinical and histopathological analysis and differential consideration of recurrent peritoneal fluid, this case turned out to be even more exciting. Microbiological nucleic acid testing with ubiquitous taeniid cestode primers revealed 99 % identity to *T. martis* (ENA accession number LT837855).

*T. martis* is a tapeworm, which dwells as the adult stage in the small intestine of carnivores. Rodents serve as an intermediate host, in which *T. martis* cysticercus larva stages mainly develop in the peritoneal and pleural cavity. In Europe, *T. martis* has been observed in martens and rodents in Italy, Germany, The Netherlands, Belgium, Spain, Poland, Belarus and Switzerland [3–5]. So far, to our knowledge, only three cases of human *T. martis* cysticercosis have been reported worldwide [6–8]. Cysticercosis manifested in the brain or in the eye as a solitary lesion. In our case, the patient also presented with a solitary lesion; however, not in the brain or eye but in the peritoneum like in the natural intermediate host reservoir. So far, a peritoneal *T. martis* cysticercosis has only been observed once in a non-human primate, in a Tonkean macaque in the Alsace region [9]. All cases displayed one unique lesion, potentially due to the non-multiplying nature of *T. martis* larvae, thus limiting the risk of generalization and recurrence. Transmission to intermediate hosts occurs via the oral route by way of food or water contaminated by faecal matter. The former two cases involved patients who were recreational gardeners, which could be a potential risk factor. No history of gardening was reported by our patient, rather extensive recreational hiking in the Alps; however, without contact with wild animals. This indicates the possibility of contamination of the environment with eggs of this tapeworm, and it is highly probable that a natural *T. martis* life cycle existed in the environment of the patient's living place with small rodents and carnivores in close vicinity.

Even though the number of diagnosed cases is low, we cannot rule out the possibility that cases may have been missed in the past due to the absence of appropriate analysis. We therefore want to stress the importance of a thorough histopathological analysis with additional molecular analysis and strongly recommend that cysticercosis of *T. martis* should be added to the differential diagnosis of unusual peritoneal tumors.
